# A Giant Verrucous Carcinoma of the Penis Presenting with Urinary Sepsis and Angina

**DOI:** 10.1155/2014/207026

**Published:** 2014-11-12

**Authors:** Michael Nomikos, Paschalis Barmpoutis, Eleni Papakonstantinou, Zacharias Chousianitis, Prodromos Ouzounoglou, Paraskevi Efstathiadou, Charilaos Katsifotis

**Affiliations:** ^1^Department of Urology, Thriassion General Hospital, Gennimata Avenue, 19200 Athens, Greece; ^2^Department of Pathology, Thriassion General Hospital, 19600 Athens, Greece

## Abstract

Penile verrucous carcinoma also known as Buschke-Löwenstein tumor in the genital region is an uncommon variant of penile carcinoma exhibiting slow, expansive growth. We present a case of a 63-year-old male who presented with a giant purulent penile mass causing urinary sepsis and angina. Regional lymph nodes were clinically negative and staging with CT scans of thorax and abdomen did not show any signs of lymph node or distant metastases. After resuscitation, radical penectomy was performed and a perineal urethrostomy was created. Histological examination revealed a Buschke-Löwenstein tumor of the penis with no invasion of corpus cavernosum and urethra.

## 1. Introduction

Verrucous carcinoma of the penis accounts for 5% to 16% of all penile squamous cell carcinomas. They are all well-differentiated low grade tumors, usually extending to the underlying stroma with a broad based pushing border. Regional lymph node metastasis is rare and distant metastases have never been reported [1]. We present a case of a 63-year-old man with a huge Buschke-Löwenstein tumor of the penis who presented with urinary sepsis and angina.

## 2. Case Presentation

**Figure 1:**
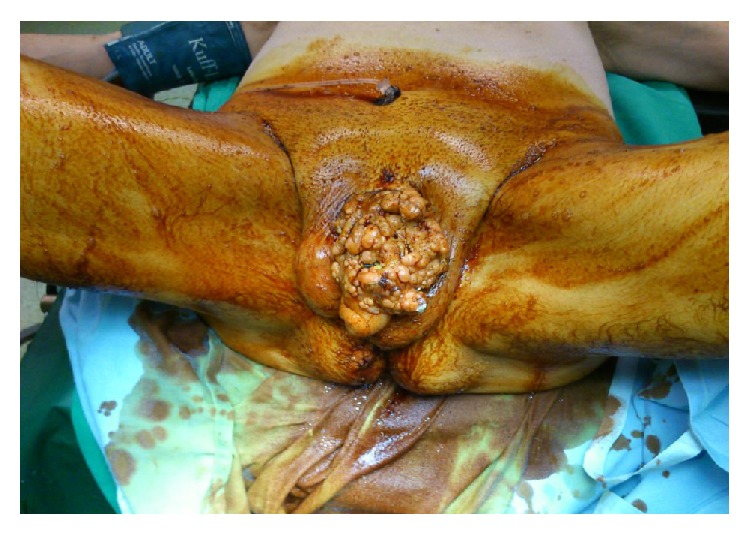
Massive cauliflower-like penile mass with several urinary fistulae making the penile shaft indistinguishable.

A 63-year-old type II diabetic man presented to the emergency department due to fatigue and chest pain. He had a temperature of 38.3°C; he was tachycardic (125 beats per minute) and hypotensive (80/60 mmHg). He also complained about dizziness and 6 kgs weight loss the last 3 months. Clinical examination revealed a massive purulent penile mass with no palpable inguinal and supraclavicular lymph nodes. The penile shaft was indistinguishable and there were several urinary fistulae with purulent discharge and a foul odour. The mass was pale pink, cauliflower-like with surface dimensions of approximately 12 × 10 cm (Figure 1).

The laboratory results showed white blood cells 23.1 × 10^3^/*μ*L, hematocrit 13.5%, and hemoglobin 4.0 g/dL, with normal urea and creatinine. Urinary culture derived from a suprapubic catheter showed* Escherichia coli* >10^5^ cfu/mL. The electrocardiogram showed ST decline suggestive of myocardial ischemia due to anemia. He was admitted and commenced on antibiotics and intravenous fluids. He was also transfused with 6 units of concentrated red blood cells and angina resolved. Three days later his hematocrit was raised to 27.3% and his hemoglobin to 8.7 g/dL. A chest CT was performed, which revealed moderate pleural effusion bilaterally. A contrast CT of the abdomen showed no lymph node invasion or distant metastases. There was also scrotal effusion. No malignant cells were present in pleural fluid analysis. An echocardiogram followed, which revealed mild pulmonary hypertension.

The results of an elective incisional biopsy of the mass were consistent with verrucous carcinoma of the penis.

He underwent radical penectomy and a perineal urethrostomy was created. The surgical specimenwas 11 cm length and 5.2 cm diameter. The urinary catheter was removed at the seventh postoperative day.

**Figure 2:**
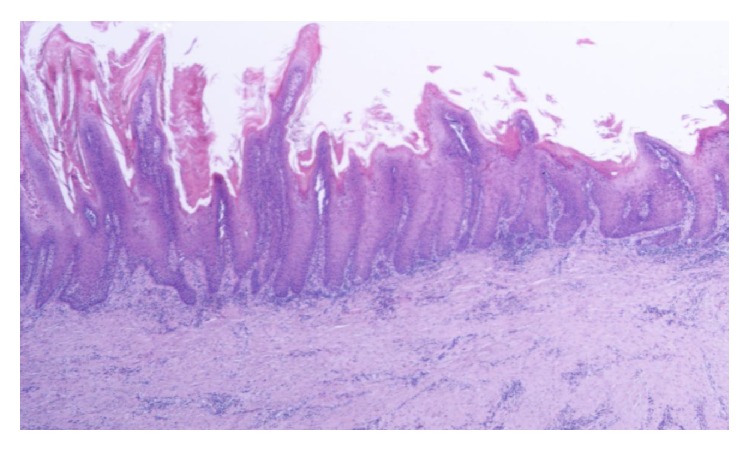
Verrucous carcinoma. Well-differentiated, broad based, papillary neoplasm with acanthosis and hyperkeratosis.

**Figure 3:**
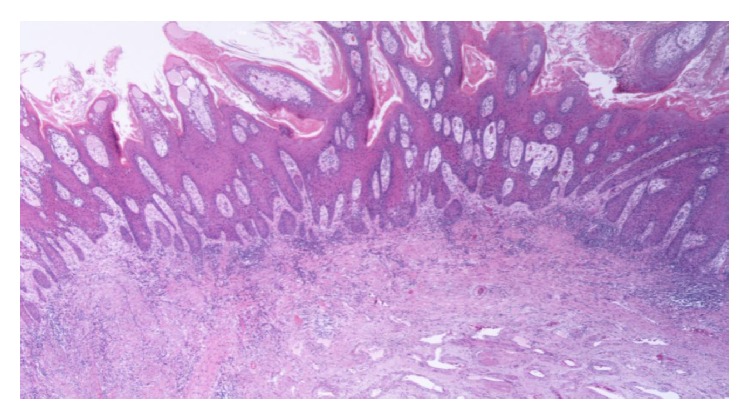
Verrucous carcinoma without invasion of the underlying corpus cavernosum.

Histological examination revealed verrucous carcinoma of the penis invading the underlying stroma with a broad based pushing border, with no invasion of the corpora cavernosa, corpus spongiosum, and the urethra. No blood vessel invasion was noted (Figures 2 and 3). The patient was discharged on the ninth postoperative day in good clinical condition. Although he was advised to, he did not receive any adjuvant therapy. Clinical examination and CT of the abdomen up to 3 years postoperatively showed no local recurrence or distant metastases and a functional perineal urethrostomy.

## 3. Discussion

Verrucous carcinoma of the penis is a rare clinical entity, first described in 1896.

In 1925, Buschke and Löwenstein further characterized the tumors as locally invasive, rapidly growing “carcinoma-like condylomata acuminata” [2]. Buschke-Löwenstein tumor is classified as a verrucous carcinoma. The term of verrucous carcinoma was first introduced by Ackerman in 1948 and was designated as a variant of squamous cell carcinoma with distinct features including well-differentiated expanding growth and verrucous appearance. It has been proposed that it represents an intermediate state between condyloma acuminatum and squamous cell carcinoma. For unknown reasons, a very small subset of long-lasting condyloma acuminatum eventually evolves into slowly invading tumors and then, if left untreated, into large papillomatous proliferations that penetrate deeply into the underlying tissue [3]. A characteristic of the tumor is its benign-appearing histological appearance, which resembles that of condyloma acuminatum. It may be difficult to distinguish between these 2 conditions, particularly at an early stage of the disease. Buschke-Löwenstein tumor is generally considered to be verrucous carcinoma in genital regions, but in some reports the lesions are regarded as distinct entities [4].   Buschke-Löwenstein tumor is characterized by local expansion, compression, and destruction of the adjacent tissues, while condyloma acuminatum always remains superficial and does not extend into adjacent tissues [5]. Ahsaini et al. recently presented a case of verrucous carcinoma arising in an extended condyloma acuminatum of the perigenital region, suggesting the theory of malignant transformation of Buschke-Löwenstein tumors if left untreated for a long time [6].

Risk factors for Buschke-Löwenstein tumors are low socioeconomic status, drug abuse, use of oral contraceptives, presence of other sexually transmitted diseases, diabetes, and smoking, which may be associated with an impaired immune response. Our patient had a low socioeconomic status and he was a heavy smoker. The etiology of verrucous carcinoma is still unknown. Human papillomavirus types 6 and 11 have been suggested to play a significant role in the pathogenesis of these tumors. However, other studies have not found a significant association between these tumors and HPV. Histologically, it may resemble a verruca superficially, with hyperkeratosis, parakeratosis, acanthosis, papillomatosis, and granular cell layer vacuolization. A characteristic feature is the blunt projections of well-differentiated epithelium surrounded by edematous stroma and chronic inflammatory cells that extend into the dermis, sometimes forming sinuses filled with keratin [7].

Clinically, penile verrucous carcinoma arises anywhere on the penis, mostly on the glans or foreskin as a gradually enlarging, exophytic, papillary, cauliflower-like, or verrucose mass which may be foul smelling and sometimes ulcerated. It evolves slowly without invasion of the corpora cavernosa or the corpus spongiosum. Regional nodal involvement or distant metastases have never reported in the literature. Although local tumour recurrence in the perianal area has reported to be up to 67% regardless of treatment method in one series, the local recurrence rate of surgically treated verrucous carcinoma of the penis is less than 5% [8]. Buschke-Löwenstein in the perianal region is more aggressive since malignant transformation has been reported in up to 50% of cases with a high recurrence rate and poor prognosis [9].

Treatment is surgical and usually extensive due to the bulky nature of these tumors. Systemic and intralesional chemotherapy has been tried, as monotherapy or as an adjunct to surgery, such as 5-fluorouracil and cisplatin. Intralesional interferon has been effective for infiltrating lesions but requires frequent treatments for a lengthy time period. Intra-aortic infusion chemotherapy of a 50 mg methotrexate every 24 hours has also been tried with the advantage of preserving the anatomical structure and sexual function in younger patients, but with controversial results. Radiation therapy remains controversial. In this case tumours behave aggressively due to radiation-like anaplastic transformation of the primary verrucous carcinoma [10, 11]. Carbon dioxide laser ablation has also been tried with promising results in younger patients with smaller lesions located in the glans. However, glansectomy should be considered the treatment of choice in patients with smaller tumours confined to the glans [12, 13].

To our knowledge, this is the first report of a giant Buschke-Löwenstein tumor of the penis presenting with unusual clinical manifestations such as urinary sepsis and angina. Radical surgical excision was the treatment of choice in our case due to his extensive local disease, with more conservative treatments preferred for smaller lesions confined to the glans. Lymphadenectomy was not performed due to significant surgical morbidity and minimal potential of lymph node metastasis. The patient was examined every three months for the first year and then every 6 months for the second and third postoperative years with no local disease recurrence and a functional perineal urethrostomy. CT of the abdomen performed the third postoperative month and then every 6 months to the third postoperative year showed no lymph node or distant metastasis.

Penile verrucous carcinoma is a rare clinical entity with locally aggressive characteristics and complete surgical management is suggested. Regional lymphadenectomy does not seem to be necessary in patients with the final diagnosis of penile verrucous carcinoma by histological examination, but close follow-up is of great importance due to substantial risk of local recurrence of the disease.
